# A three-dimensional ex vivo model recapitulates in vivo features and drug resistance phenotypes in childhood acute lymphoblastic leukemia

**DOI:** 10.1038/s41375-025-02739-8

**Published:** 2025-09-10

**Authors:** Magdalini Kanari, Iria Jimenez Garcia, Fabio D. Steffen, Lisa A. Krattiger, Charles Bataclan, Wangjie Liu, Benjamin R. Simona, Bart Deplancke, Olaia Naveiras, Martin Ehrbar, Beat Bornhauser, Jean-Pierre Bourquin

**Affiliations:** 1 University Children’s Hospital Zurich, Pediatric Oncology and Children’s Research Center, Zurich, Switzerland; 2 University Hospital Zurich, Department of Obstetrics, Zurich, Switzerland; 3 Laboratory of Regenerative Hematopoiesis, Department of Biomedical Sciences, University of Lausanne, Lausanne, Switzerland; 4 Laboratory of Systems Biology and Genetics, Institute of Bio-Engineering & Global Health Institute, School of Life Sciences, EPFL, Lausanne, Switzerland; 5 ECTICA Technologies AG, Zurich, Switzerland; 6 Swiss Institute of Bioinformatics, Lausanne, Switzerland; 7 Hematology Service, Departments of Oncology and Laboratory Medicine, Lausanne University Hospital (CHUV), Lausanne, Switzerland

**Keywords:** Preclinical research, Leukaemia

## Abstract

Acute lymphoblastic leukemia (ALL) preferentially localizes in the bone marrow (BM) and displays recurrent patterns of medullary and extra-medullary involvement. Leukemic cells exploit their niche for propagation and survive selective pressure by chemotherapy in the BM microenvironment, suggesting the existence of protective mechanisms. Here, we established a three-dimensional (3D) BM mimic with human mesenchymal stromal cells and endothelial cells that resemble vasculature-like structures to explore the interdependence of leukemic cells with their microenvironment. This model recapitulates recurrent topologic differences between B-cell and T-cell precursor ALL, whereby B-ALL interacts more closely with the mesenchymal compartment. Migration versatility was found to be associated with subtype, consistent with increased motility observed in T-ALL in vivo. Single-cell RNA signatures revealed similarities to profiles from in vivo patient derived xenografts, suggesting relevant states ex vivo. Furthermore, enhanced migration, adherence and cell cycle heterogeneity was visualized in our co-culture model. Finally, drug response experiments in this 3D model confirm clinically relevant sensitivity and resistance patterns that reflect specific disease phenotypes and may provide a broader dynamic range for drug response testing.

## Introduction

Acute lymphoblastic leukemia (ALL) is the most common pediatric cancer originating from the malignant transformation of lymphoid progenitor cells in the bone marrow (BM) with frequent involvement of medullary and extra-medullary sites [1]. During leukemogenesis, the BM forms a protective ecosystem which contributes to the propagation and acquired chemoresistance of ALL cells leading to relapse [2–4]. The non-hematopoietic compartment, including mesenchymal stromal cells (MSCs), osteo-lineage cells, endothelial cells (ECs), fibroblasts, chondrocytes and adipocytes, plays a substantial role in hematopoiesis and has been directly linked to the transformation of the BM into a tumor supporting niche [5, 6]. Increased vascularization, adhesive interactions with ECs, MSCs and osteoblasts through expression of CXCR4 (CXC motif chemokine receptor 4), VLA-4 (very late antigen-4) and CD44 (homing cell adhesion molecule) molecules as well as hypoxia induction are amongst common alterations in the leukemic BM [7]. Fibroblast-driven extracellular matrix (ECM) remodeling, reduced bone density by impaired osteoblast function, fatty acid consumption and adipocytic employment for chemoresistance further sustain this leukemic niche [8, 9]. We have previously shown by using in vivo patient-derived xenograft (PDX) models that residual leukemia cells can survive chemotherapeutic stress in the BM microenvironment without clonal selection or additional mutations, suggesting the presence of protective cues in this niche [10]. Nonetheless, these molecular signaling events have thus far remained elusive yet will represent candidates for interference to eradicate resistant populations.

Two-dimensional co-culturing systems support leukemic expansion but lack the hierarchical complexity and cell communication that occurs in the BM [11]. In recent years, three-dimensional models have emerged as a promising tool for the study of leukemia to identify underlying interactions with its niche as well as to predict drug response more accurately by recapitulating the microenvironment protection [12]. Different approaches have been implemented including scaffold-based systems like hydrogels, spheroids, organ-on-a-chip applications, or microfluidic devices [13–22]. In all of the studies, the authors describe leukemia-stromal interactions, modulated gene expression and phenotypic plasticity as well as increased chemoresistance induced by supporting microenvironmental cues. Patient-derived MSC spheroids support the expansion of leukemic samples and validate the presence of a leukemic subpopulation that mimics minimal residual disease signatures [14]. 3D microfluidic chips, resembling the in vivo morphology and cellular composition of the BM, have demonstrated dynamic interactions between cellular types and linked microenvironment-promoted quiescence to increased drug resistance [13, 15]. However, these systems are complex, difficult to reproduce at scale and lack general applicability for patient-derived leukemic models.

In this study, we developed a 3D BM model compatible with high-content screening in 96-well imaging plates. The three component system consists of human MSCs and ECs that form 3D blood vessel-like structures, readily providing a niche for culturing patient-derived ALL cells. We identified distinct ALL features in the presence of the supporting cells and identified topological patterns resembling in vivo localization. We performed single-cell RNA sequencing (sc-RNAseq) of all cellular compartments to identify molecular cues that underlay the leukemic-microenvironment interactions and phenotypically and functionally characterized 13 leukemic PDXs in this system. Finally, we investigated whether drug resistance is driven by the 3D support and compared it with two-dimensional (2D) response in order to identify potential enhanced predictive accuracy of drug testing.

## Results

### A 3D vasculature-like model for ALL patient-derived xenografts

**Fig. 1:**
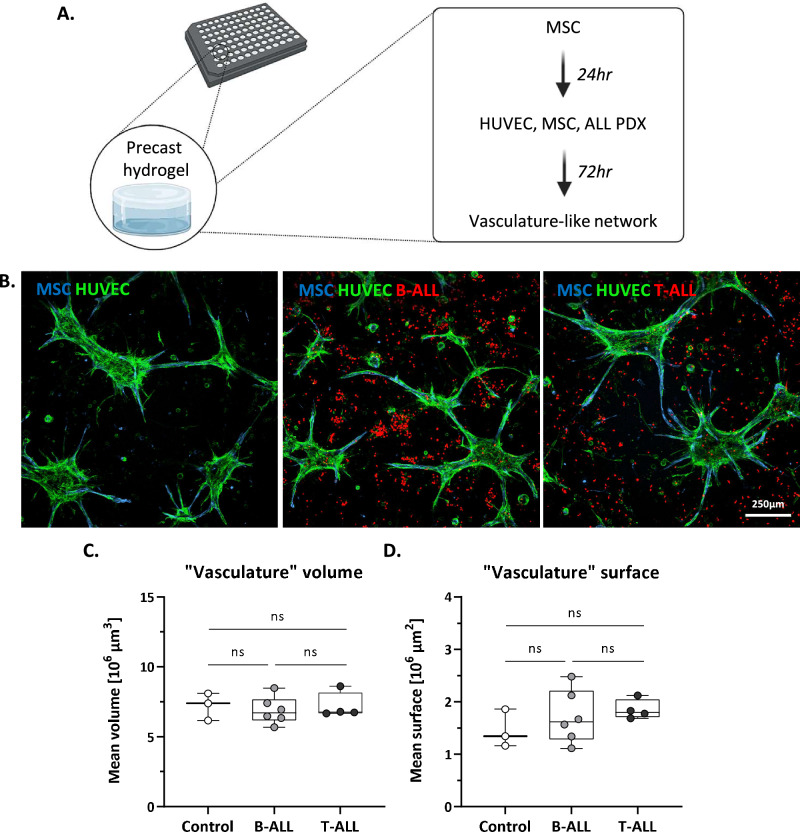
Description of the 3D vasculature-like model for leukemic PDXs. **A** Experimental set-up. The 3DProSeed® Hydrogel Well Plate was used for the establishment of the ex vivo model. Primary MSCs are cultured for 24 h, followed by the addition of HUVECs, MSCs and ALL PDXs. **B** Representative confocal fluorescence images of the co-cultures in maximum intensity projections using confocal z-stacks (z-step: 10 μm) showing MSCs (blue), HUVECs (green), and ALL cells (red). **C**, **D** A vasculature-like network is detected in a similar manner in all 11 conditions tested (MSC-HUVEC controls, 10 PDXs co-cultures with MSC and HUVECS). Depicted are the (**C**) mean volume and (**D**) mean surface of the HUVECs (“vasculature”) in triplicates. Controls were quantified in triplicates in three separate experiments (statistical analysis: unpaired *t* test, significant *p* value < 0.05).

To evaluate the role of the non-hematopoietic BM in the leukemic ecosystem, we developed a 3D model that mimics blood vessel-like structures, enabling functional studies of their interactions with leukemic cells. This was achieved by co-culturing stromal, endothelial and leukemic cells in 96-well imaging plates containing pre-cast, optically transparent synthetic hydrogels. These hydrogels are based PEG-peptide bioconjugates and contain adhesion and degradation motives supporting cell spreading, MMP-mediated gel degradation and formation of cell-produced ECM. Moreover, an in-depth surface density gradient allows the characterization of motility patterns for different cell types (Fig. 1A) [23–27]. The primary human BM MSCs are necessary for endothelial spreading, acting as a supporting scaffold (Supplementary Fig. 1A), while no “vascularization” occurs in their absence (Supplementary Fig. 1B). Interestingly, the hTERT-MSC (human telomerase reverse transcriptase-MSC) cell line is not capable of supporting this “vascularization”, highlighting the importance of using primary MSCs for a blood-vessel mimic (Supplementary Fig. 1C). 3D segmentation and characterization of the structures revealed consistent volumes and surfaces of network amongst the control, an MSC-HUVEC co-culture, and MSC-HUVEC-ALL co-cultures established with 10 PDXs, thus validating the robustness of the model across multiple conditions (Fig. 1B–D).

### Leukemic cells exhibit enhanced motility upon immediate interaction with the supporting cells

**Fig. 2:**
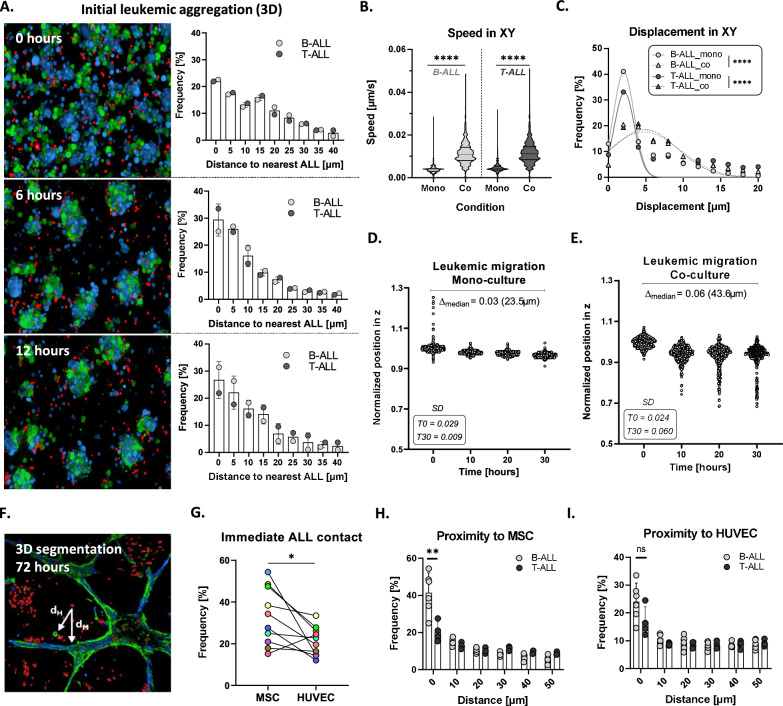
ALL PDX cells exhibit distinct phenotypes upon immediate interaction with microenvironment cells. **A** Maximum intensity projections of confocal z-stacks (z-step: 7.8 μm) showing the immediate formation of multicellular clusters consisting of MSCs (blue), HUVECs (green) and ALL cells (red) at 0 h, 6 h and 12 h upon seeding. The leukemic cells follow the clustering behavior of MSCs and HUVECs towards the network formation (12 h, 5 min interval). The corresponding bar plots show decreased ALL cell-to-cell distances, quantified as the minimum 3D distances between two leukemic cells (3D single-cell analysis, bin center = 5). **B**, **C** Motility analysis of segmented ALL single cells: (**B**) speed (**C**) displacement (bin center = 2, statistical analysis: Kolmogorov-Smirnov unpaired t tests with *p* < 0.0001). **D**, **E** Image-based single-cell segmentation and quantification of the vertical positioning (z-axis) of ALL cells, normalization based on the median position of each timepoint. Data obtained by 30-h live cell imaging with a 5 min interval. Depicted is the Δ in median z-position from timepoint 0 to 30 based on the normalized data (and actual values in μm), as well as the standard deviation (SD) at 0 (T0) and 30 (T30) hours. **F** 3D segmentation of all cell types at 72 h enables proximity analysis of ALL cells relative to MSCs or HUVECS, dH: minimum ALL distance to HUVEC, dM: minimum ALL distance to MSC. **G** Percentage of ALL cells located within <10 μm of MSC and HUVEC (statistical analysis: paired Wilcoxon signed rank test with *p* < 0.05, *n* = 3 per condition, 10 different PDXs as biological replicates). **H**, **I** Topological analysis of ALL cells stratified by molecular subtype. **H** B-ALL PDXs are significantly closer to MSCs than T-ALL PDXs, while (**I**) no subtype-dependent difference is observed in proximity to HUVECs (unpaired *t*-test with Welch’s correction, *p* < 0.05; *n* = 3 per condition).

To analyze the influence of the microenvironment on leukemic cell behavior, we performed timelapse confocal imaging directly after seeding for 30 h with a timestep of 5 min, comparing 3D ALL mono-cultures and ALL co-cultures with MSCs and ECs. It is evident that the leukemic PDXs directly interact with the supporting cells as they form multi-cellular clusters over time. Cell-to-cell distance analysis by single cell segmentation quantification revealed enhanced leukemic aggregation over time, with decreasing ALL to ALL cell distances in a 12-h course (Fig. 2A). Significantly higher speed and displacement in the XY plane was observed for the leukemic cells in co-culture compared to mono-culture, as measured in the first 6 h of the timelapse imaging (Fig. 2B, C). The movement of the ALL cells in the z-axis was also influenced by the presence of supporting cells. ALL cells in mono-culture remained concentrated around the median position, with decreasing standard deviation from 0 to 30 h, indicating no substantial migration (Fig. 2D). In contrast, in co-culture we observed an increased migratory behavior of the leukemia cells with a subpopulation exhibiting increased penetration capacity, as indicated by the increased values of both delta median and standard deviation at time 30 compared to time 0 (Fig. 2E).

Furthermore, the positioning of the leukemic cells towards MSCs and HUVECs was evaluated after 72 h, where the network formation is completed. Full-volume segmentation and proximity analysis of all cell types was performed and the minimum distance of each ALL to the nearest respective cell type (dH: ALL distance to HUVEC, dM: ALL distance to MSC) was calculated (Fig. 2F). Increased 3D proximity to MSCs compared to HUVECs was observed for 9/10 of the PDXs analyzed, with the exception of one T-ALL PDX residing closer to HUVECs, while the localization towards the two cell types was not directly correlated (Fig. 2G). When stratified by molecular subtype, B-ALL PDXs showed markedly closer proximity to MSCs than T-ALL PDXs, while no significant differences were observed in ALL cell distances to HUVECs (Fig. 2H,I). These findings suggest that B-ALL cells exhibit a subtype-specific topological bias toward mesenchymal stromal cells within the leukemic niche model. No significant difference in spatial localization could be detected between molecular subtypes across the B-ALL and T-ALL PDXs (Supplementary Fig. 1D, E).

### MSCs exhibit multi-lineage differentiation potential in the blood-vessel mimic, resembling in vivo cell fate

To better understand the heterogeneity amongst ALL PDX samples and their associated bone marrow-like microenvironments, we performed single-cell RNA sequencing (scRNA-seq) to decipher the transcriptomic signatures of these distinct phenotypes. The conditions included all the cell types (MSC, HUVEC, ALL) cultured alone, an MSC-HUVEC control and 6 PDX leukemic co-cultures (MSC-HUVEC-ALL, referred as PDX1-PDX6 based on the leukemic PDX per condition) of different ALL molecular subtypes. With the use of established cell surface markers (CD19, and CD7 for B- and T-ALL, THY-1 for MSC and CDH5 for HUVEC) for their identification, both supporting and leukemic cells were analyzed by clusters.

**Fig. 3:**
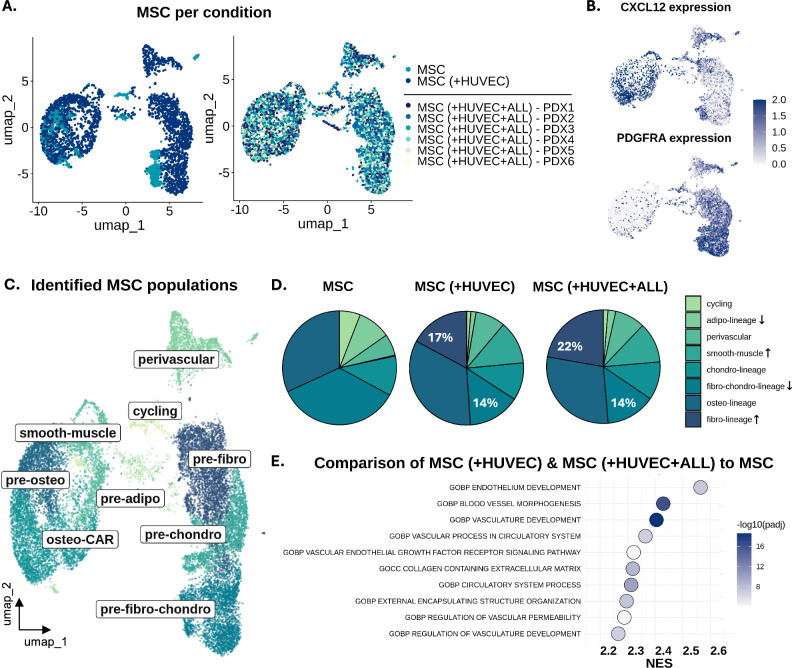
MSC multi-lineage differentiation in 3D resembles in vivo and human data. **A** UMAP projections showing MSC population overlap based on the culturing condition; left: two controls, i.e. MSC and MSC ( + HUVEC), right: MSC ( + HUVEC + ALL), PDX1-PDX6 indicating the six different ALL PDXs). **B** Feature plots depicting the expression of CXCL12 (top) and PDGFRA (bottom) across the MSC populations. **C** Identification of multi-lineage MSC subpopulations based on established lineage-specific gene signatures, projected in UMAP, upon coculturing in 3D. **D** Proportional distribution of MSC subtypes across conditions, with the increased population percentages in white. Arrows indicating increase or decrease upon co-culture. **E** GSEA dot plot showing the top 10 enriched pathways in MSCs from the MSC( + HUVEC) and MSC( + HUVEC + ALL) to the MSC control comparison.

Enhanced MSC differentiation was observed in the presence of HUVECs, both in the control (MSC-HUVEC) and leukemic conditions (MSC-HUVEC-ALL) compared to the MSC control, and well-established gene sets were used to identify the different MSC clusters (Fig. 3A, Supplementary Fig. 2A) [3, 28–34]. Notably, a polarization based on the known MSC-markers PDGFRA (platelet-derived growth factor receptor alpha) and CXCL12 (CXC motif chemokine 12) was observed, consistent with in vivo and human primary MSCs, with PDGFRA-MSCs exhibiting high multipotent capacity and CXCL12-MSCs preferentially committed to osteo-lineages (Fig. 3B) [35–38]. Unsupervised clustering revealed nine MSC subpopulations, including precursors of osteo-, adipo-, fibro- and chondro-lineages, with some cells characterized by the co-expression of genes related to more than one lineage. Other clusters included cycling MSCs with high MKI67 expression and more committed populations such as perivascular and smooth muscle cells (Fig. 3C, Supplementary Fig. 2B-D). Moreover, increased differentiation towards smooth muscle cells and pre-fibroblasts was observed, potentially due to their capability of producing vascular promoting and supporting factors such as the vascular endothelial growth factor (VEGF) [39, 40]. In the leukemic conditions (MSC-HUVEC-ALL), a modest enrichment (5%) in the fibro-lineage is detected, possibly describing the transition into a cancer-associated fibroblastic phenotype that facilitates the homing of ALL cells close to the network (Fig. 3D) [41]. Gene set enrichment analysis (GSEA) suggests that MSCs support vascularization when co-cultured with endothelial cells, consistent with findings from a previous 3D co-culture leukemia model [14]. Specifically, blood vessel morphogenesis and endothelium development were among the most upregulated pathways, with ECM-remodeling or pro-angiogenic genes such as COL5A3, COL4A1, MMP9, TGFB1, KDR and FLT1 being enriched (Fig. 3E). Notably, when comparing MSCs from the MSC-HUVEC-ALL co-cultures to MSCs cultured alone, pathways related to external encapsulating structure organization are enriched, while not in the MSC-HUVEC control, suggesting leukemia-specific remodeling of the stromal matrix (Supplementary Fig.2E, F). Finally, cell-cell communication network analysis revealed known supporting interactions between MSCs and ALL, with the highest probability being through CD74 and CD44 signaling (Supplementary Fig. 2G).

### GSEA indicates EC-induced ECM reorganization in the presence of leukemic cells

**Fig. 4:**
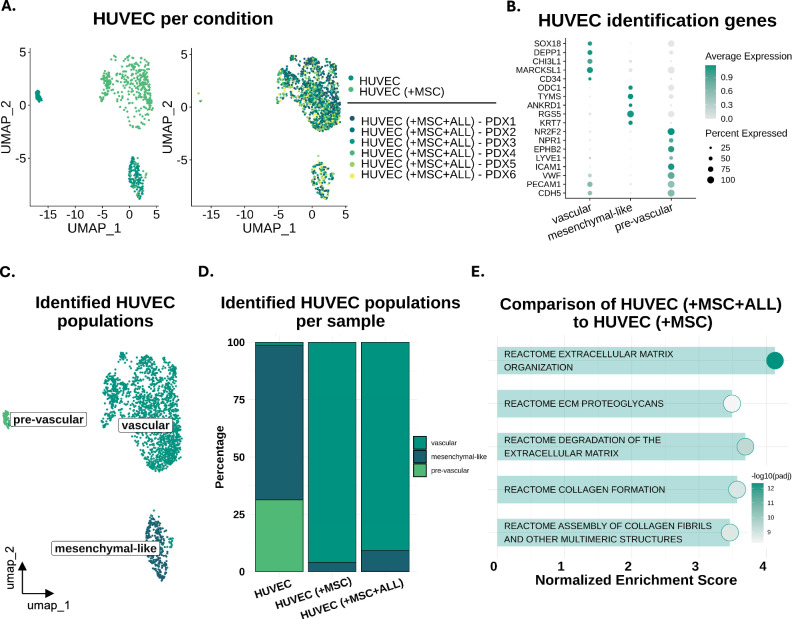
Endothelial cells re-organize the microenvironment in the leukemic conditions. **A** UMAP projections showing HUVEC population overlap based on the culturing condition; left: two controls, i.e. HUVEC and HUVEC ( + MSC), right: HUVEC ( + MSC + ALL, PDX1-PDX6 indicating the six different ALL PDXs). **B** Dot plot depicting the expression of representative marker genes used to identify transcriptionally distinct HUVEC subpopulations. **C** UMAP projection with the corresponding HUVEC subpopulations. **D** Stacked bar plot depicting the relative abundance of each HUVEC subpopulation per condition. **E** GSEA dot plot showing degradation of the ECM amongst the top 5 pathways enriched in the HUVECs cultured in MSC-HUVEC-ALL co-cultures.

On the other hand, ECs exhibit less complexity but still consist of distinct subpopulations including a mesenchymal-like, a pre-vascular and a vascular cluster (Fig. 4A–C), with a notable transition to the vascular-like phenotype observed in all co-cultures (Fig. 4D, Supplementary Fig. 2H) [28, 42, 43]. Another significant observation is the enrichment of the extracellular matrix degradation pathway, and other ECM-related ones, exclusively in the leukemic conditions (MSC-HUVEC-ALL co-cultures), while in the control (MSC-HUVEC) only enhanced proliferation is observed when comparing to the HUVEC control (Fig. 4E, Supplementary Fig. 2I). This could indicate that endothelial cells degrade and re-organize the ECM upon leukemic infiltration in order to create a favorable niche, as they are already known regulators of ECM dynamics [26].

### Leukemic gene expression in the 3D microenvironment mirrors signatures detected in xenografts in vivo

**Fig. 5:**
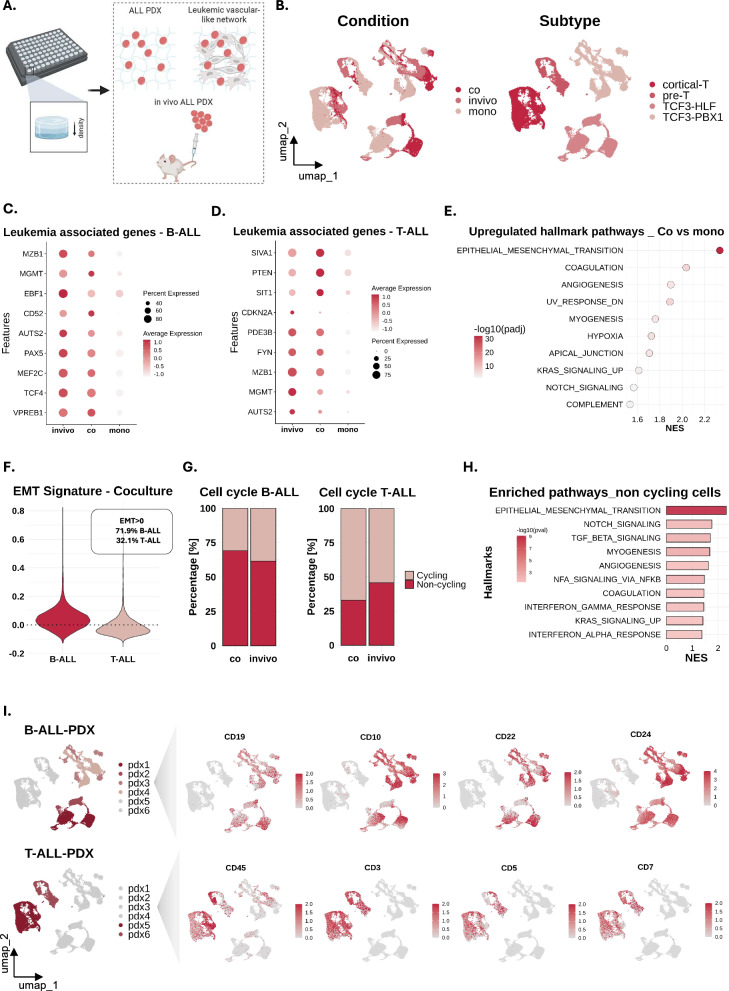
Comparison of leukemic gene expression from 3D co-culture and in vivo leukemia xenografts. **A** Sequencing of leukemic PDXs in three different conditions, mono-, co-culture and bone marrow cells from leukemia xenografts in vivo. **B** UMAP projections of the integrated datasets (RPCA reduction through Seurat), visualized by condition (left) and leukemia subtype (right). Subtype is the leading source of variability, determining the clustering in the UMAP projection. **C** Expression of leukemia-associated genes for B-ALL (scaled dot plot) across conditions. **D** Expression of leukemia-associated genes for T-ALL (scaled dot plot) across conditions. **E** GSEA comparing co-cultured ALL cells to mono-cultured. Depicted are the top 10 upregulated hallmark pathways. EMT is revealed as the most upregulated pathway in co-cultures. **F** Violin plot showing the expression of the EMT signature expression in B- and T-ALL cells for the co-culture, revealing higher EMT activity in B-ALL. **G** Stacked bar plots showing the proportion of cycling and non-cycling cells in B- and T-ALL for the two conditions, co-culture and in vivo. **H** GSEA bar plot depicting the top 10 upregulated pathways comparing non-cycling (G1) to cycling cells (G2M and S). **I** UMAP projections depicting the B-ALL (top, pdx1-pdx4) and T-ALL (bottom, pdx5-pdx6) PDXs, linked to expression of known ALL cell surface markers.

To identify potential differences in the transcriptome of leukemia cells in mono- and in co-culture, we performed scRNA-seq of 6 different ALL PDXs under both conditions. In order to capture potential subtype heterogeneity, 4 different ALL subtypes were selected including two TCF3::HLF positive B-ALLs, two TCF3::PBX1 B-ALLs, one cortical T-ALL and one pre-T-ALL. In addition, these PDXs were also extracted and sequenced directly from the murine bone marrow (referred to as in vivo) with the aim of identifying potential similarities between those conditions (Fig. 5A). All the cells were successfully integrated and clustered based on the ALL subtype (Fig. 5B, Supplementary Fig. 3A). Differential gene expression analysis (DGEA) revealed upregulation of genes that are associated to B- and T-cell development as well as leukemogenesis when comparing co-cultured to mono-cultured ALLs. The corresponding dot plots highlight the similar expression of these genes in vivo and in co-culture, suggesting that the ex vivo model recapitulates essential features for leukemic development (Fig. 5C, D).

Further comparison revealed that genes regulating migration, adhesion and ECM-remodeling are amongst the most upregulated genes in co-cultured ALL (Supplementary Fig. 3B). The epithelial-mesenchymal transition (EMT) hallmark, described by some of the previously mentioned genes, was found to be the top pathway enriched, while the overall regulatory mechanism of EMT was consistently upregulated as well (Fig. 5E, Supplementary Fig. 3C). Notably, in recent studies the EMT pathway has been associated with increased aggregation and invasiveness in the context of leukemia, reminiscent of the enhanced motility upon immediate ALL-microenvironment interaction we have observed (Fig. 2A–E) [44–48]. Independent analysis of B- and T-ALL cells validated EMT enrichment in both co-cultured subtypes, while this signature was predominant in B-ALL with approximately 70% overall expression (Fig. 5F, Supplementary Fig. 3D, E). An EMT signature was also detected in PDX that were isolated from the murine bone marrow, more prominently in B-ALL compared to T-ALL (Supplementary Fig. 3F). B-ALL has been described by enhanced microenvironment interactions in vivo compared to T-ALL, in line with its dependency on an EMT-like phenotype [10]. Moreover, association of cell cycle arrest and EMT-like leukemic populations has also been described [47]. Cell cycle analysis revealed that the 3D co-cultures successfully reiterate in vivo subtype-based proliferation heterogeneity with similar proportions of cycling and non-cycling cells; however, GSEA revealed that the EMT pathway is actually predominantly regulated by the non-cycling cells, potentially confirming a connection between cell cycle arrest and the EMT leukemic traits (Fig. 5G,H, Supplementary Fig. 3G). Finally, subpopulation heterogeneity based on known cell surface markers was detected, with cells exhibiting various expression profiles across the same PDX (Fig. 5I).

### Preserved cell heterogeneity in co-culture is validated by single cell microscopy and flow cytometry

**Fig. 6:**
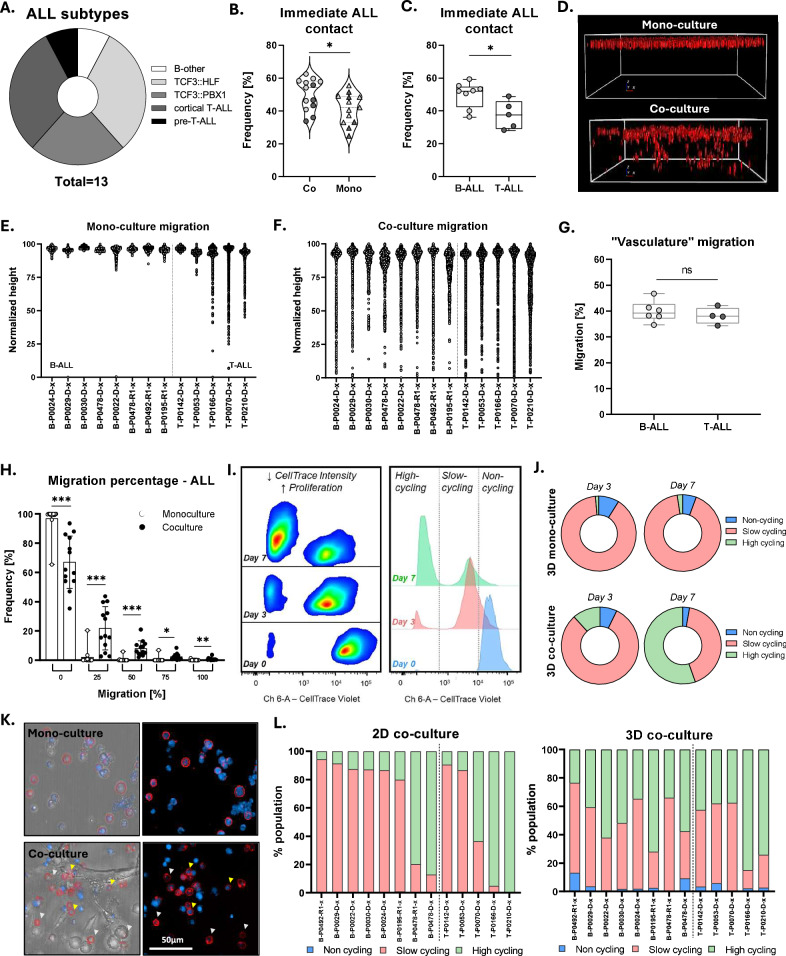
3D co-culture-enhanced ALL aggregation, migration and proliferation lead to increased heterogeneity. **A** Pie chart showing that 13 PDXs of different subtypes were analyzed in the 3D model. **B** Immediate cell-cell proximity ( < 10 μm) is enriched in co-cultures, suggesting superior cell communication (*n* = 3, PDXs used as biological replicates, statistical analysis: Wilcoxon matched-pairs signed rank test, *p* value < 0.05). **C** Immediate contact is significantly enriched in B-ALL samples compared to T-ALL (same statistics as B). **D** Representative 3D reconstruction of B-ALL cells in the hydrogel. **E, F** Single-cell spatial distribution normalized to the highest ALL z-position per well. Light gray: B-ALL PDXs, Dark gray: T-ALL PDXs. **G** Dot plot showing the migration percentage of HUVECs, normalized to the highest leukemic cell position detected in the hydrogel (*n* = 3, statistical analysis: unpaired *t* test, *p* value < 0.05). **H** Migration analysis reveals that approximately 20–40% of ALL cells are in direct contact with the vessel-like structure (*n* = 3, statistical analysis: Wilcoxon matched-pairs signed rank test, *p* value < 0.05). Migration percentages are based on the relative position of each cell to the highest localized ALL cell. **I** Proliferation assessment with CellTrace Violet via Flow Cytometry. The CellTrace intensity reveals a non-cycling (defined by timepoint 0, high CellTrace intensity), a slow cycling (medium CellTrace intensity) and a high cycling population (low CellTrace intensity). **J** Plots depicting the percentage of cells in each phase across the 13 PDXs per condition. **K** Immunofluorescent staining with CD19 for lymphocyte (B-ALL) identification. Both CellTrace positive (yellow arrows, non-cycling) and negative cells (white arrows, cycling) are detected. **L** Stacked bar plots depicting the proportions of non-cycling, slow cycling and high cycling cells in 3D and 2D co-culture respectively on day 7.

In order to investigate whether an EMT-like, i.e. aggregated and migratory, phenotype is indeed present in the co-cultures, phenotypic and functional characterization was performed. For that purpose, thirteen PDXs of different subtypes including TCF3::HLF, TCF3::PBX1, B-other and T-ALL were seeded in both mono- and co-culture and analyzed using confocal microscopy and flow cytometry after 72 h (Fig.6A). Single-cell distance analysis revealed increased aggregation in the co-culture, with the majority of the leukemic cells maintaining close distances of approximately 10 μm (Fig. 6B, Supplementary Fig. 4A). Subtype-based analysis revealed enhanced clustering in B-ALL compared to T-ALL, associating B-ALL to the observed EMT increase in our transcriptomic data (Fig. 6C, Fig. 5F). In addition, migration of the ALL cells in the hydrogel was promoted by the non-hematopoietic cells when compared to mono-cultures, where minimum migration is detected and only a subset of T-ALL cells exhibited enhanced migratory capacity, yet again confirming T-ALL versatility (Fig. 6D, E). On the other hand, migration is observed in the case of all co-cultures (Fig. 6F). The portion of leukemic cells, in direct contact with the vasculature-like network, was assessed based on the HUVECs migration capability, with the network settling at a depth of 40% distance from the top (with the highest leukemia cell as position reference) regardless of the ALL subtype used (Fig. 6G). No leukemia cells were found in this depth in mono-cultures, while 20–40% of the co-cultured ALLs were able to migrate closely to the network, irrespective of the subtype (Fig. 6H, Supplementary Fig. 4B, C).

Furthermore, the leukemic PDXs were stained with CellTrace Violet and their proliferation was monitored for 7 days (Fig. 6I, Supplementary Fig. 4D). On day 3, no significant difference is observed across the conditions or subtypes, as expected based on the transcriptomic data (Fig. 6J, Supplementary Fig. 4E–G). However, at a later timepoint (day 7), increased proliferation is observed in the co-culture, showing beneficial conditions for leukemia’s growth. Interestingly, a portion of the leukemic cells decrease or pause their cellular growth rate, validating the existence of a non-cycling population as well as proving that the 3D co-culture maintains the cell cycle heterogeneity that is observed in in vivo studies (Fig. 6J). These findings were also validated by immunofluorescence, where upon CD19 staining for the lymphocyte identification, the presence of both CellTrace positive (yellow arrows, non-cycling) and negative cells (white arrows, cycling) was evident (Fig. 6K). Nonetheless, no specific localization based on the cycling state was observed, similarly to our previous in vivo observations where, upon chemotherapy, no selective cell death occurs based on the proliferation phase of the cells (Supplementary Fig. 4H–K) [10]. Finally, comparison of the proliferation rate of the ALL PDXs to the standard 2D hTERT MSC-ALL co-culture system revealed preserved cell state heterogeneity in 3D. Leukemic cells present a distinct proliferation phenotype in 2D with patient samples either strongly proliferating or completely lacking division capacity, while in 3D both cycling and non-cycling populations are detected for all PDXs on day 7, thus highlighting that the 3D model can recapitulate cellular complexity ex vivo when 2D systems fail (Fig. 6L).

### Drug response patterns are reproduced, with increased resistance in 3D

**Fig. 7:**
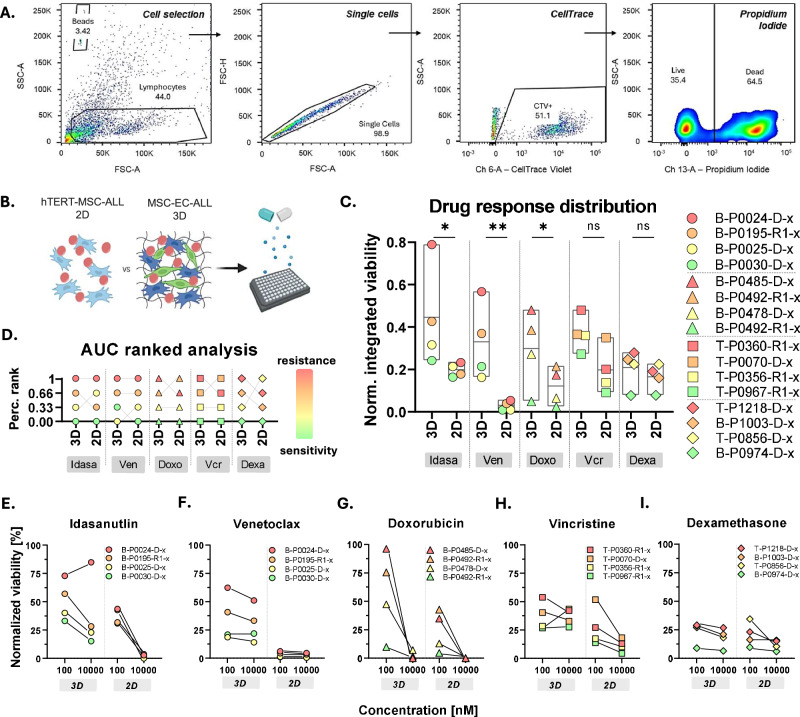
Drug screenings in the 3D co-culture reveal increased resistance. **A** Gating strategy for flow cytometry. Viable ALL cells were gaited for lymphocytes based on their size, followed by doublet exclusion, cellTrace positivity and PI negativity. **B** Experimental design. Drug responses in the 3D co-culture system were compared to those in a standardized drug response profiling protocol, where immortalized hTERT-MSCs-ALL co-cultures are treated for 72 h (*n* = 3). **C** Bar plots showing the normalized response values (integrated normalized cell counts over two concentrations) for all the 3D and 2D conditions (*n* = 3, statistical analysis: ratio paired t test, *p* value < 0.05). **D** Percentile ranking indicating the response patterns across the conditions, color-coded based on the response in 3D. **E**–**I** Dot plots showing the normalized viability of the leukemic cells upon treatment with different compounds. Depicted is the response to 100 and 10,000 nM in the 2D and 3D systems respectively. From left to right : (**E**) idasanutlin, (**F**) venetoclax, (**G**) doxorubicin, (**H**) vincristine, and (**I**) dexamethasone.

Finally, we investigated whether the 3D co-cultures can be utilized for drug response evaluations and if the distinct phenotypes observed alter drug sensitivity. Flow cytometry was performed to evaluate the 3D response. For that purpose, 16 PDXs of various subtypes were treated for 72 h, with compounds of interest specifically for each subgroup, and then enzymatically extracted for evaluation. Flow cytometry analysis was performed by gating for CellTrace positive cells, i.e. lymphocytes, based on unstained controls, followed by propidium iodide gating for the exclusion of dead cells (Fig. 7A). The viability of the ALL cells was compared to 2D controls that were treated in the same manner and analyzed by high throughput microscopy (Fig. 7B).

The predictive value of the 3D model was validated as the samples were following the response patterns of the established 2D drug response profiling (DRP) platform (Fig. 7C). Percentile ranking was performed to confirm the sensitive/resistant samples of each cohort (Fig. 7D). Remarkably, there was increased viability in 3D upon treatment for the majority of the PDXs (Fig. 7E–I). 14 out of 20 samples (70%) showed decreased sensitivity (>10% difference in normalized cell counts to DMSO) at 100 nM for the 3D co-cultures, suggesting superior ALL protection upon treatment. This phenotype can be attributed to multiple factors, as the presence of both ECM and ECs have been linked to leukemic treatment escape [26, 49, 50]. In addition, a remaining subpopulation can be observed at 10 μM for 4 drugs (exception doxorubicin) indicating the existence of a resistant subpopulation that cannot be detected in 2D (Fig. 7E–I). Finally, a comparative analysis of the steroid response in 2D and 3D was performed for 8 ALL patients with known poor response during the steroid prephase of the AIEOP-BFM-ALL treatment protocol (Clinical information, Supplementary Table 2). Consistent with our observations, significantly increased normalized viability was observed in 3D for poor response ALL patients (Supplementary Fig. 5A). This difference was also evident through normalized integrated viability assessment (integrated normalized cell counts over two concentrations) for both B- and T-ALL samples (Supplementary Fig. 5B). However, further validation of the comparative predictive value of 2D and 3D responses using clinically annotated samples, as well as the inclusion of additional chemotherapeutic agents, is required.

Taken together, this model recapitulates established drug response patterns thus can be used for compound testing applications, while the decreased drug efficacy observed could also potentially lead to the identification of resistance patterns more readily compared to 2D.

## Discussion

Three-dimensional bone marrow models have demonstrated significant improvements in the study of leukemia, as they incorporate essential niche elements including various cellular types, ECM communication and mechanical support; however, they mostly focus on the study of acute myeloid leukemia (AML) and not ALL [51]. In this study, a 3D hydrogel-based BM mimic for ALL PDXs is presented, characterized by robustness, reproducibility and compliance with high-throughput readouts. The model recapitulates blood-vessel like structures by culturing human primary MSCs and ECs with the ALL PDXs in a responsive hydrogel that facilitates cell-cell and cell-ECM communication. Subtype-dependent localization towards the vasculature-like structures was observed, with B-ALL exhibiting closer contact with stromal or endothelial cells whilst T-ALL displays less preferential localization to cellular structures. Barz et al. demonstrated similar phenotypes in an in vivo xenograft model with B-ALL cells residing in proximity to vascular and endosteal niches in the BM, while T-ALL cells exhibited increased versatility [10]. This highlights the capability of our ex vivo model to reproduce subtype-relevant interaction features observed in vivo.

The BM microenvironment impact on leukemic progression is well-described; however, whether the niche acts as a predisposition factor for disease or it is transformed into a favorable environment upon crosstalk with malignant cells is yet unclear [52]. Single-cell transcriptomic profiling of the 3D cultures revealed multi-lineage differentiation of the MSCs in the vasculature-like system, with a polarization based on the CXCL12 and PDGFRA markers, linking the ex vivo phenotype to the ones described in human or murine models with distinct lineage commitment [35–38]. Smooth muscle cells and fibroblasts were defined as the predominant lineages in the HUVEC-containing conditions, potentially due to their capability of producing vascular-promoting and supporting factors [39, 40]. No significant ALL-induced alterations were observed suggesting that longer culture duration may be required to capture the dynamics of their crosstalk. Conversely, HUVECs upregulated the ECM degradation pathway under leukemic influence, suggesting a microenvironment-driven reorganization of the extracellular matrix that may facilitate malignancy, consistent with findings by Verma et al., who showed that ECM degradation contributes to leukemic progression [53]. Thus, this ex vivo vasculature-like model can also be utilized to determine trajectory fates of supporting cells, and to identify key mediators of their interdependence with leukemia.

The relevance of the co-culture system to leukemic in vivo features was demonstrated by the comparable expression of necessary B- and T-cell developmental genes to those found in PDX cells isolated directly from the murine BM. Notably, these genes were not significantly expressed when ALL cells were cultured alone in the hydrogel, highlighting the essential role of the microenvironment components for a leukemic 3D model, as shown in other studies for AML [51]. Furthermore, through further analysis of the leukemic profile in 3D co-culture, the EMT pathway was identified as the most highly enriched. EMT-like leukemia has been described by increased invasiveness in multiple studies, with Park et al. recently showing that MSC-adherent ALL exhibits enhanced survival and resistance to treatment compared to non-adherent cells through overexpression of EMT-related genes [44–48]. Single-cell topological analysis revealed enhanced aggregation and migration in the 3D co-culture. Interestingly, only a distinct subpopulation (20–40% of ALL cells) exhibited these properties thus highlighting preserved heterogeneity in the BM mimic. Subtype-based variability was detected with B-ALL exhibiting increased dependency on the niche, with enhanced aggregation in co-culture as well as limited migration capability when cultured alone compared to T-ALL. Cell cycle arrest has also been associated to the EMT-like phenotype and chemoresistance [47]. Proliferation analysis revealed both rapidly and slowly cycling cells, preserving cell state heterogeneity that our 2D model was incapable of. Taken together, the 3D BM mimic recapitulates transcriptomic signatures and reflects subpopulation heterogeneity comparable to that observed in the bone marrow in vivo.

3D models are increasingly used to study disease and to validate therapeutic targets, as they provide additional layers to better recapitulate in vivo complexity [22, 54]. Comparing the drug response phenotypes of a relatively simple 3-D system to the Drug Response Profiling (DRP) platform enabled the identification of phenotypes that would not have been predicted by genomic information and which we evaluate in clinical research to inform personalized therapies [55–57]. The model captures differences in sensitivity and resistance that reflect patient characteristics. Moreover, the range of sensitivity and resistance detected in 3D was broader than in the 2D DRP while reflecting similar patterns comparing different ALL PDXs. In the 3D system, resistant leukemic subpopulations were identified at the maximal drug concentration of 10 μM for 4 out of 5 drugs tested. This is in line with reports that suggest that the enhanced complexity of leukemic 3D models provides niche support conferring increased drug resistance, which may improve detection of resistant subpopulations in this assay [13, 15, 58–60]. It is also possible that the 3D system modifies special drug distribution in the context of both the ECM-mimic and the ECs, which is challenging to verify [61]. Further correlation of ex vivo 3D DRP data to in vivo responses in ALL xenografts and in the clinic would be required to demonstrate the value of our model.

Additionally, expanded analysis incorporating a larger representation of ALL genomic subtypes would be necessary to confirm the capability of our approach to capture and characterize ALL heterogeneity under chemotherapeutic pressure at the single cell level. scRNA-seq upon treatment could help capture the complexity of ex vivo responses, while comparison with in vivo treated PDXs using our established murine PDX model could identify patterns of minimal residual disease. We have already shown that in vivo disease signatures can be recapitulated in our ex vivo model. If signature similarities are maintained upon treatment as well, we will potentially have a tool to assess treatment options and study resistance patterns, reducing the need for murine models.

Further refinement of 3D image analysis will be essential to scale up the approach and provide comprehensive topological and phenotypic characterization of ALL PDXs, ultimately complementing and potentially replacing flow cytometry-based drug response readouts. Identifying resistance patterns linked to cellular position, proximity to niche components, or phenotypic alterations will offer new therapeutic avenues by targeting leukemia-microenvironment interactions. In addition, these advances will support the use of this system for high-content drug combination screening and functional genetic studies.

Taken together, we have established a 3D vasculature-like model that reproducibly mimics physiological-like features. We have shown that drug responses can be studied in this system, systematically recapitulating 2D response patterns for each PDX, with increased resistance, thus potentially allowing the identification of relapse-driving populations. This 3D co-culture system is readily adaptable for implementation, as all required components, including the relevant cell types and 96-well plates, are commercially available. The hydrogel plates are robust and allow for reproducible assays, as demonstrated in our study. Nonetheless, in contrast to the well-established 2D DRP, clinical implementation of this model, particularly in high-throughput, is limited by the number of parallel perturbations, imaging time and complexity, computational resources and increased costs. Moreover, translation into clinical research applications will require stringent quality control and standardization procedures to ensure data consistency and regulatory compliance. Such systems can bridge the gap between in vivo and ex vivo analyses and potentially lead to profound understanding of different mechanisms governing disease progression, microenvironment interactions and therapy resistance.

## Materials and methods

### Patient-derived xenografts

The primary patient samples were collected through bone marrow aspiration upon written informed consent of the patients’ parents or legal guardians, in accordance with the Declaration of Helsinki. The ethics commission of the Canton of Zürich granted the approval for the sample collection with the approval number 2014-0383. The primary samples were then injected (fresh or frozen) via intravenous injection in immunodeficient NSG (NOD.Cg-*Prkdcscid IL2rgtm1Wjl*/SzJ) mice, that were purchased from The Jackson Laboratory. The human leukemic engraftment was monitored weekly by flow cytometry and xenografts were recovered by samples enrolled in the ALL-BFM 2000 and 2009 and ALL-REZ BFM 2002 studies.

### Cell culturing

The primary human mesenchymal stromal cells (MSC) were provided by Prof. Dr. Martin Ehrbar. The extraction of the MSCs through bone marrow aspiration of healthy donors was previously described in Papadimitropoulos et al. 2014 [62]. The cells were cultured in MEMa (22571-020, ThermoFisher) supplemented with 10% FBS (S0615, Sigma), 1%PS (15140122, ThermoFisher) and 5 ng/mL FGF-2 (PHG0369, ThermoFisher) at 37°C with 5% CO2.

The Human Umbilical Vein Endothelial Cells were also provided by Prof. Dr. Martin Ehrbar, purchased from Angioproteomie (PELO Biotech). The cells were cultured in EGM-2 (CC-3162, Lonza) supplemented with 10% FBS in collagen-coated (A1048301, ThermoFisher) flasks at 37°C with 5% CO_2_. Both unlabeled (PB-CH-190-8011) and GFP-transduced (PB-CAP-0001GFP) HUVECs were used for the formation of vascular-like networks.

### 3D culturing

The 3D co-culture was established in the 3DProSeed 96-well hydrogel plate (ECT.PS1.001.096, Ectica Technologies), a black, glass-bottom imaging plate with 96 wells containing pre-cast, synthetic, animal-free, and optically transparent PEG-based hydrogels. Cell suspensions were prepared in MEMα supplemented with 10% FBS, 1% PS, and 5 ng/mL FGF-2. The hydrogel storage buffer was aspirated from the plate and discarded, and 200 μL of cell suspension was added to each well, followed by incubation at 37°C with 5% CO₂. For the MSC-HUVEC-ALL co-culture, 30,000 primary human MSCs were seeded per well initially. After 24 h, 30,000 HUVECs, 30,000 ALL cells, and 15,000 MSCs were added to each respective well. MSC-HUVEC co-cultures (MSC were incubated for 24 h, followed by seeding of HUVEC and MSC) and ALL monocultures served as controls, under the same experimental conditions. Vascular-like network formation required 72 h following the addition of HUVECs. For long-term cultures (7 days), an additional 100 μL of medium was added on the third day.

### Hydrogel digestion

Cells were seeded as described above (3D culturing). At the digestion timepoint, the supernatant of each well was collected and 200 uL of digestion mix was added. The mix included Liberase 0.2 mg/mL (5401119001, Sigma) DNase 200 U/mL (11284932001, Sigma) in MEMa. The mix was incubated for 30 min at 37°C with 5% CO_2_, with a manual mixing step at 15 min. The cell suspension was collected and 200 uL of MEMa was added for the dilution of the digestion mix. The samples were centrifuged at 300 g for 5 min and resuspended in the appropriate solvent.

### Cell staining

CellTrace agents i.e. CellTrace Violet, CellTrace Yellow and CellTrace FarRed (C34571, C34573, C34572, ThermoFisher) were used for the staining of the different cellular types for sequential microscopy analyses. The staining of the cells followed the manufacturer’s instructions, whereas the cells were stained for 20 min in PBS at RT and washed for 5 min, followed by resuspension in medium and seeding.

### Immunostaining

Anti-human CD90 conjugated to FITC or APC (11-0909-42, ThermoFisher) were used for the staining of the MSCs. Anti-human CD31 conjugated to FITC or Pacific Blue (557508 BD biosciences, 303114 Biolegend) were used for the HUVECs. 1:300 of antibody was directly added in the medium of the well and incubated for 30 min. For the leukemic cells, antibodies were selected based on the subtype, with CD19 APC (345791, BD Bioscience) for B-ALL and CD7 PE (12-0079-42, Thermofisher) for T-ALL. 1:600 of antibody was directly added in the medium of the well and incubated for 2 h and 30 min.

### Confocal microscopy

High-content confocal microscopy was performed with an Operetta CLS (PerkinElmer). The objectives used were 10x, 20x and 40x with z-stack acquisition. The standard step size for 10x imaging was 7.8 um. All conditions were imaged in triplicates. Time-lapse imaging was performed with 10x magnification, 5 min time interval and 10 um step size for the z-stack acquisition.

### Confocal microscopy analysis

All microscopy analysis was performed with Harmony (version 4.9), the Operetta CLS software. All of the images were loaded either as a 3D reconstruction or a maximum projection (based on the requirement of the downstream analysis) and each cellular type was segmented with the appropriate algorithm model. For the calculation of 3D volume and surface, the *find image region* function is implemented and the segmentation mask is created based on the pixel intensity in relation to the local or absolute threshold. The choice of the threshold depends on the fluorophore used. For the calculation of the position in z, the *find nuclei* function is performed for the 3D segmentation of leukemic cells, while for the other cell types *find image* region is selected as above, and the *calculate position properties* building block results in the quantification of the centroid’s position of each single cell/region in the well. Following the same principle, the cross population properties (minimum distance to nearest neighbor) are identified by calculating the distance from the selected object’s border to the object border of the nearest neighbor object. Finally, 2D motility analysis is performed through the *calculate kinetics properties* building block, with current step size calculated by (S_-1_ + S_+1_) and current speed by (s_-1_ + s_+1_) / (t_+1_ + t_-1_), with s as the position and t as the corresponding time point.

### Proliferation analysis

ALL cells were stained with CellTrace Violet and the seeding was performed as described above (3D culturing). After 3 and 7 days, the hydrogels were digested and the cell suspensions were resuspended in 100uL PBS each. 1uL of 7-AAD was added in each sample and left for 5 min, for further exclusion of dead cells. Flow Cytometry experiments were conducted at a BD LSRFortessa (BD Biosciences) and all flow cytometry data were analysis using the FlowJo software (version 10.8.1).

### In vivo experiments

Tail intravenous injection of ALL patient-derived xenografts into unconditioned 6 to 8-week-old mice according to animal care regulations after approval by legal authorities (125/2013,124/16, 131/19) was performed for the PDX expansion. The level of human leukemia was monitored weekly by peripheral blood sampling. The blood samples were stained with anti-human CD45 Alexa Fluor 647, CD19 PE or CD7 PE(304018 Biolegend, 302208 Biolegend, 12-0079-42 Thermofisher) and anti-mouse CD45 eFluor450 (48-0451-82, Invitrogen) with 1:100 dilution and analyzed with flow cytometry for the engraftment calculation.

### Sample preparation for single-cell RNA sequencing

For the 3D samples, cells were seeded as described above (3D culturing). After 72 h, the hydrogels were digested and cell suspensions were collected. For the sample preparation, the protocol for the fixation of cells for chromium fixed RNA profiling (Chromium Next GEM Single Cell Fixed RNA Sample Preparation Kit, 1000414, 10x Genomics) was followed based on the manufacturer’s instructions. Briefly, the samples were fixed for 24 h at 4 °C, counted with an automated cell counter EVE^TM^ Plus (NanoEntek) with the use of trypan blue and resuspended in glycerol (G5516, Sigma) for long term storage.

For the in vivo samples, the femurs were harvested and the leukemic cells were flushed through the bone marrow and cryopreserved. Upon thawing, the samples were sorted using a BD FACS Aria to ensure leukemic population purity, with the use of CD19 PE (302208, Biolegend) for B-ALL and CD7 PE (12-0079-42, Thermofisher) for T-ALL immunostaining. Samples were stained with trypan blue and counted manually with a hematocytometer. Finally, samples were resuspended in PBS containing 0.04% BSA.

### Library construction for single-cell RNA sequencing

Samples were multiplexed (1000456 for ex vivo samples and 1000261 for in vivo samples, 10x Genomics) and pooled to a total of 10000 cells per run. Then, they were loaded and sequenced with the use of an Illumina platform. The standard library protocol for 10x genomics single cell RNA sequencing for fixed and fresh cells respectively was followed based on the manufacturer’s instructions. 59,931 cells (35,545 leukemic) were recovered for the ex vivo samples, while 7,707 leukemic cells for the in vivo and were further processed computationally.

### Single-cell RNA data analysis

The processing of the raw sequence data was performed with CellRanger, using the reference genome library hg19.The data were analyzed with the R software (version 4.1.2) according to the standard pipeline described in the Seurat package (version 5.0.3). The pre-processing includes filtering of low-quality cells, doublet exclusion using the DoubletFinder package (version 2.0.3) and data integration using the RPCA reduction through the Seurat tool for the leukemic ex vivo and in vivo datasets (no integration was needed for either stromal or endothelial cell as all sequencing was performed in one batch). Further analysis included gene set enrichment analysis (GSEA package, version), cell cycle scoring and identification of inter-cellular communication patterns (CellChat package, version 2.1.2). All *p* values > 0.05 were considered significant. Further information can be found in the published code (code availability).

### Drug treatment

The compounds vincristine, doxorubicin, dexamethasone, venetoclax and idasanutlin (S1241, S1208, S1322, S8048, S7205; Selleckchem) were directly added to the well supernatant with the use of the D300 drug dispenser (Tecan) after 72 h of culture. The concentrations used were 100, 1000 and 10,000 nM, depending on the experimental conditions. The drugs were incubated for 72 h, followed by a microscopy (Operetta CLS, PerkinElmer) or flow cytometry (BD LSRFortessa, BD Biosciences) readout.

The flow cytometry readout included digestion of the hydrogels as described above. The samples were resuspended in 100uL PBS. 1uL propidium iodide (P1304MP, ThermoFisher) was used per sample for 5 min to determine the dead cells. For the viability quantification, 5uL AccuCount fluorescent counting beads (ACFP-70-10, Spherotech) were added per sample. All conditions were normalized to DMSO controls (10uM, D8418, Sigma).

### Two-dimensional drug response profiling

Two-dimensional drug response profiling followed the established protocol from DRP Zurich, described in Frismantas et al. [55]. 2500 h-TERT MSCs in AIMV (C35012, ThermoFisher) were plated in a 384 well plate. After 24 h, 10,000 leukemic cells in AIMV were seeded on top of the MSCs for the establishment of co-cultures. The following day, the drug compounds were added using the D300 drug dispenser (Tecan) at the same concentrations as above for 72 h. For the viability determination, CyQuant staining (CyQUANT Cell Proliferation Assay, C35012, ThermoFisher) was performed for 45 min (1:40 dilution for suppressor, 1:600 dilution for compound, MEMa).

### Statistics

All statistical analyses were performed using the software GraphPad Prism (Version 10.0.2). *P* values < 0.05 were considered significant. The statistical tests performed were chosen based on each data set and are indicated at the figure legend of the corresponding graph.

## Supplementary information

Supplementary figures 1-5 and supplementary tables 1-2

## Data Availability

The data supporting the findings of this study are available from the corresponding author upon request to J.-P.B. The single-cell RNA sequencing data can be found on GEO (accession numbers GSE282806, GSE284242).

## Code availability

The code used for the analysis can be found on GitHub: https://github.com/leukemia-kispi/Kanari-Leukemia-2025.
