# Supplementary material for: A three-dimensional ex vivo model recapitulates in vivo features and drug resistance phenotypes in childhood acute lymphoblastic leukemia
Source: Leukemia. 2025 Sep 10;39(12):2881–94. doi: 10.1038/s41375-025-02739-8 (PMC12634434; doi:10.1038/s41375-025-02739-8)
Supplement: Supplementary file 1 — Supplementary figures 1-5 and supplementary tables 1-2 [file 41375_2025_2739_MOESM1_ESM.pdf]

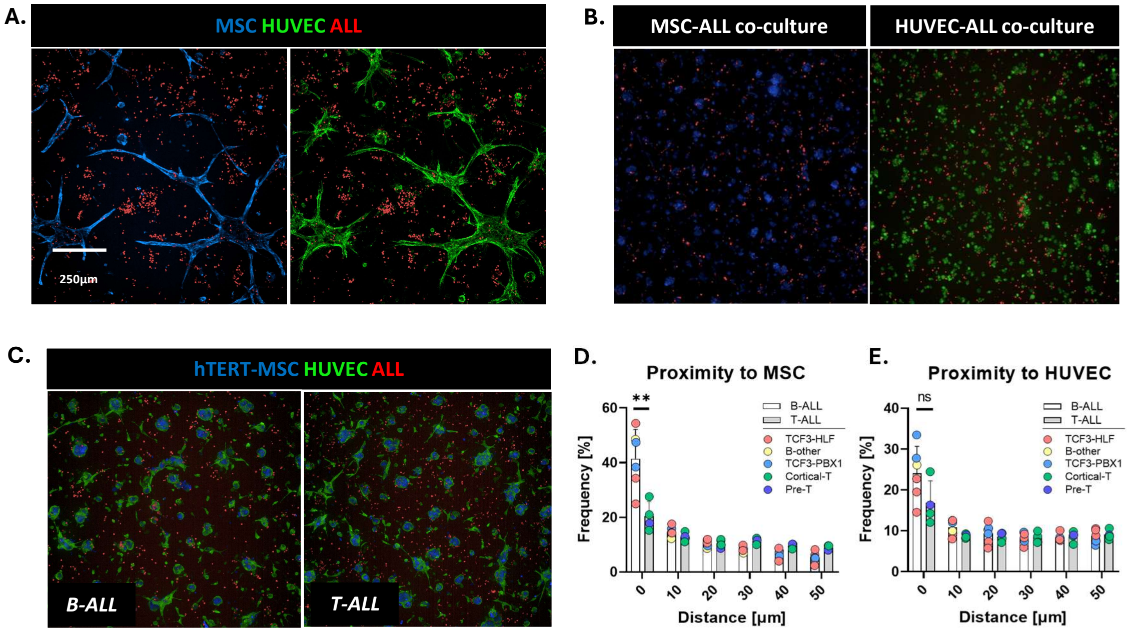

Suppl.Fig.1: "Vascularization" occurs only in the case of coculturing MSC and EC cells and in the presence of primary stromal cells. A. Maximum projections of 3D cocultures (z-step size 10µm), left depicting MSC, right HUVEC and ALL in both (MSC in blue, HUVEC in green, ALL in red). B. Maximum projection of 3D cultures. Leukemic cells (red) were cocultured with MSCs (blue, left) and HUVECs (green, right) separately. No network can be formed in either condition as the MSCs act as a scaffold for the network formation while no vascular-like network can be formed in the absence of endothelial cells. C. Immortalized hTERT-MSCs were cocultured with HUVECs and leukemic PDXs with the described protocol (3-D culturing). Upon 72 hours, maximum projections show no network formation, further reinforcing the observation that primary cells are needed to recapitulate physiological-like conditions. D,E. Comparison of topological ALL features towards MSCs and HUVECs respectively, color-coded based on the molecular subtype (bin center = 10).

A.

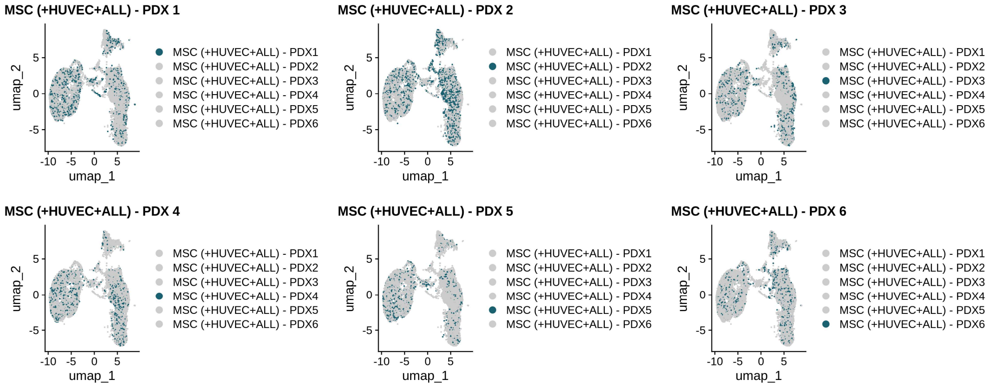

B.

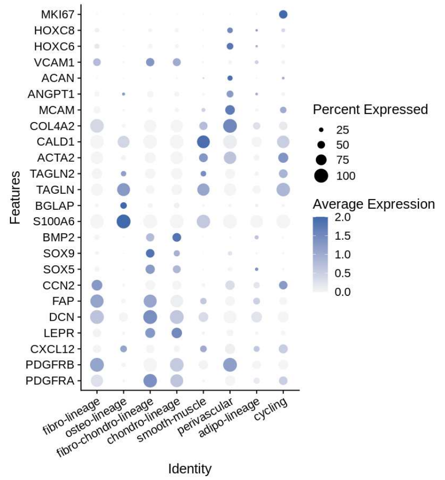

C.

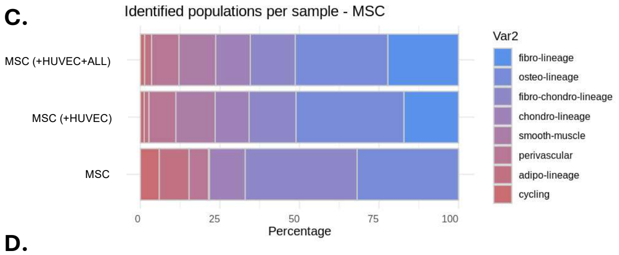

D.

	MSC	MSC (+HUVEC)	MSC (+HUVEC+ALL)
fibro-lineage	0.0	17.2	22.2
osteo-lineage	31.9	33.9	29.1
fibro-chondro-lineage	35.2	14.7	14.1
chondro-lineage	11.3	10.7	10.9
smooth-muscle	0.3	12.4	11.5
perivascular	6.1	8.5	8.6
adipo-lineage	9.4	1.5	2.2
cycling	5.9	1.2	1.3

E.

F.

G.

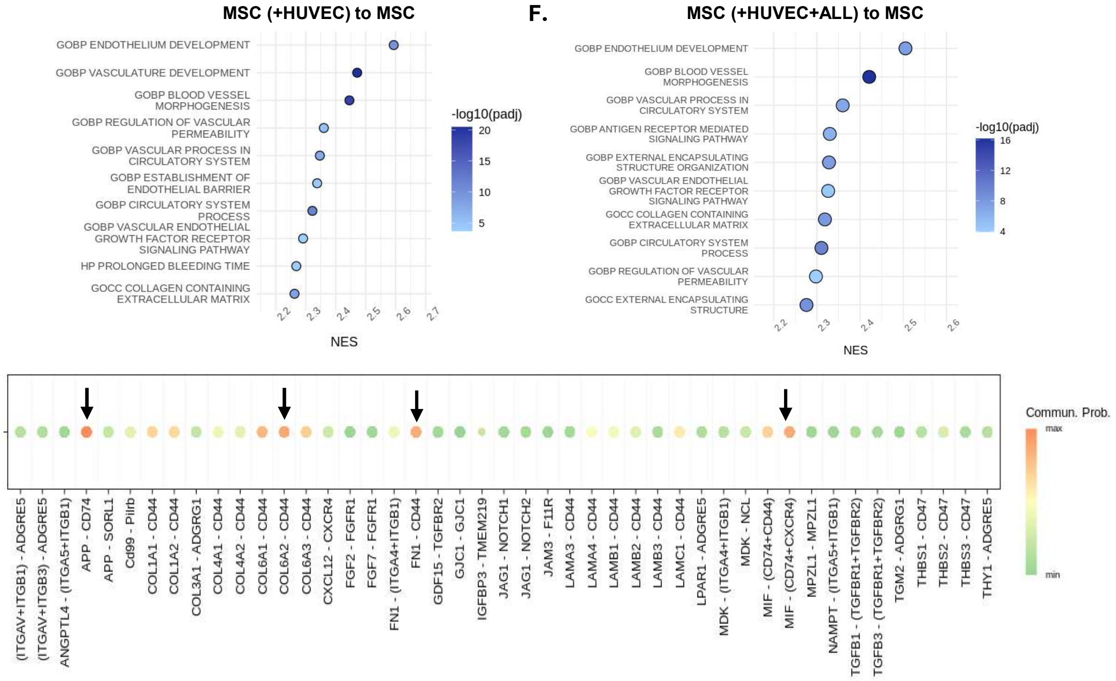

H.

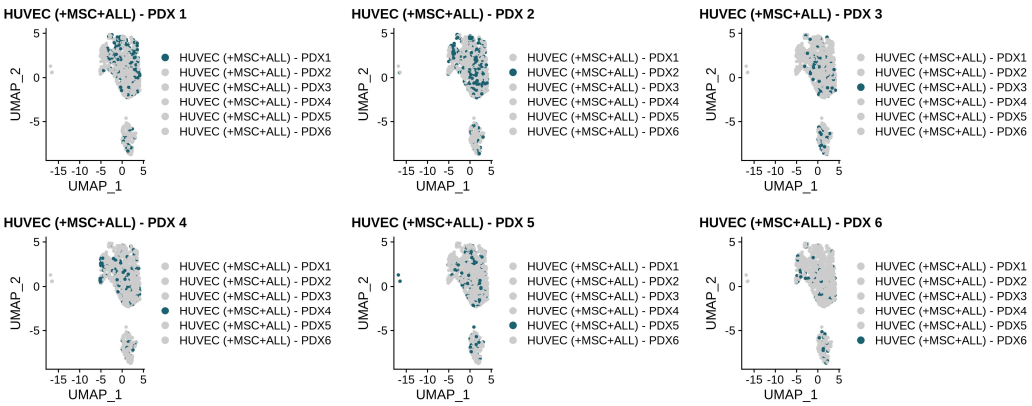

I.

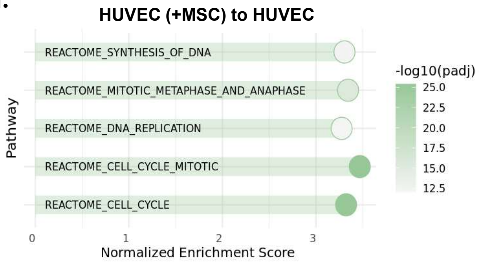

Suppl. Fig.2: Supplementary material for the supporting cells. A. Individual UMAP projections of the MSCs cultured with HUVECs and ALLs, based on the PDX used for each condition (PDX1-PDX6 corresponding to the 6 different ALL PDXs. B. MSC subpopulations. Dot plot showing the expression of known MSC markers that are used for the identification of distinct subpopulations. PDGFRA, PDGFRB, CXCL12 and LEPR expression is shown as they are considered known MSC markers with distinct expression. C. Stacked bar plot depicting the population distribution, as percentages, across the experimental conditions [MSC, MSC (+ HUVEC), MSC (+HUVEC + ALL)]. D. Table showing the percentages of the MSC subpopulations across the conditions. E,F: GSEA comparing the conditions separately. Left: MSC (+HUVEC) vs MSC, right: MSC (+HUVEC+ALL) vs MSC. G: Analysis of cell-cell communication networks through the CellChat package. Bubble blot depicting the interaction axes between MSC and ALL, with the communication probability. Arrows indicating the most probable communications. H. Individual UMAP projections of the HUVECs cultured with MSCs and ALLs, based on the PDX used for each condition (PDX1-PDX6 corresponding to the 6 different ALL PDXs. I. GSEA comparing HUVEC (+MSC) vs HUVEC. Pathways related to HUVEC replication are upregulated in the vascular condition, validating phenotypic observations as HUVEC display very low viability when cultured alone in the hydrogel.

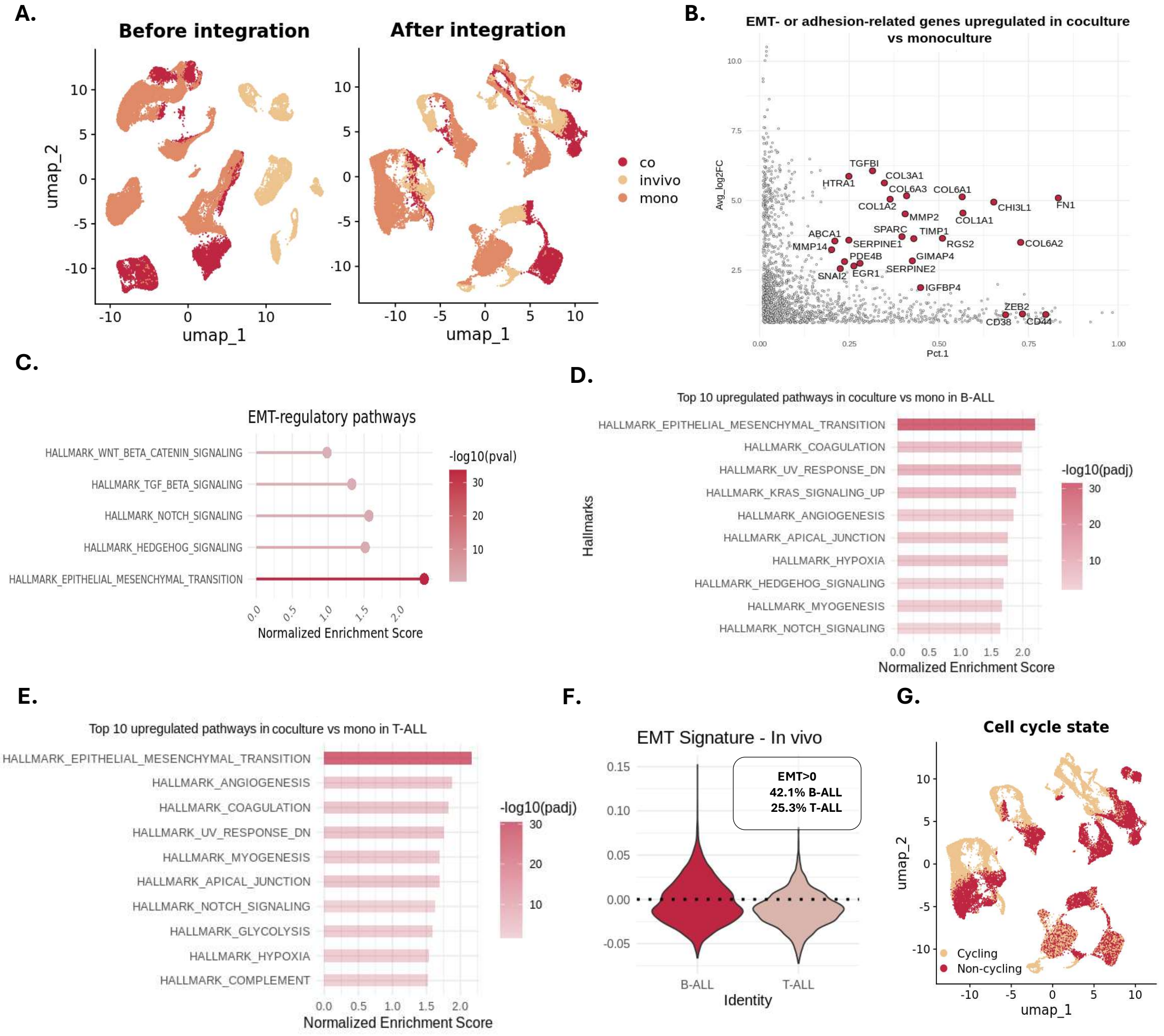

Suppl.Fig.3: Supplementary materials for leukemic PDXs. A. UMAP projections of the ALL PDXs before (left) and after (right) integration. The conditions are initially separated based on the sequencing batch, with co- and mono-cultures clustering together. Upon integration based on the condition, the samples successfully integrate based on the leukemic subtype. B. Bubble plot showing the upregulation of EMT-related genes in co-cultures C. GSEA revealed that all the pathways the regulate the epithelial-mesenchymal transition pathway are upregulated in co-culture when comparing to mono. D,E. Subtype-based GSEAs showing that the EMT pathway is independently upregulated in co-culture for both B- and T-ALL. F. Violin plots depicting the expression of the EMT signature in the PDX cells that were isolated directly from the murine bone marrow. G. Cell cycle analysis plotted on the UMAP projection.

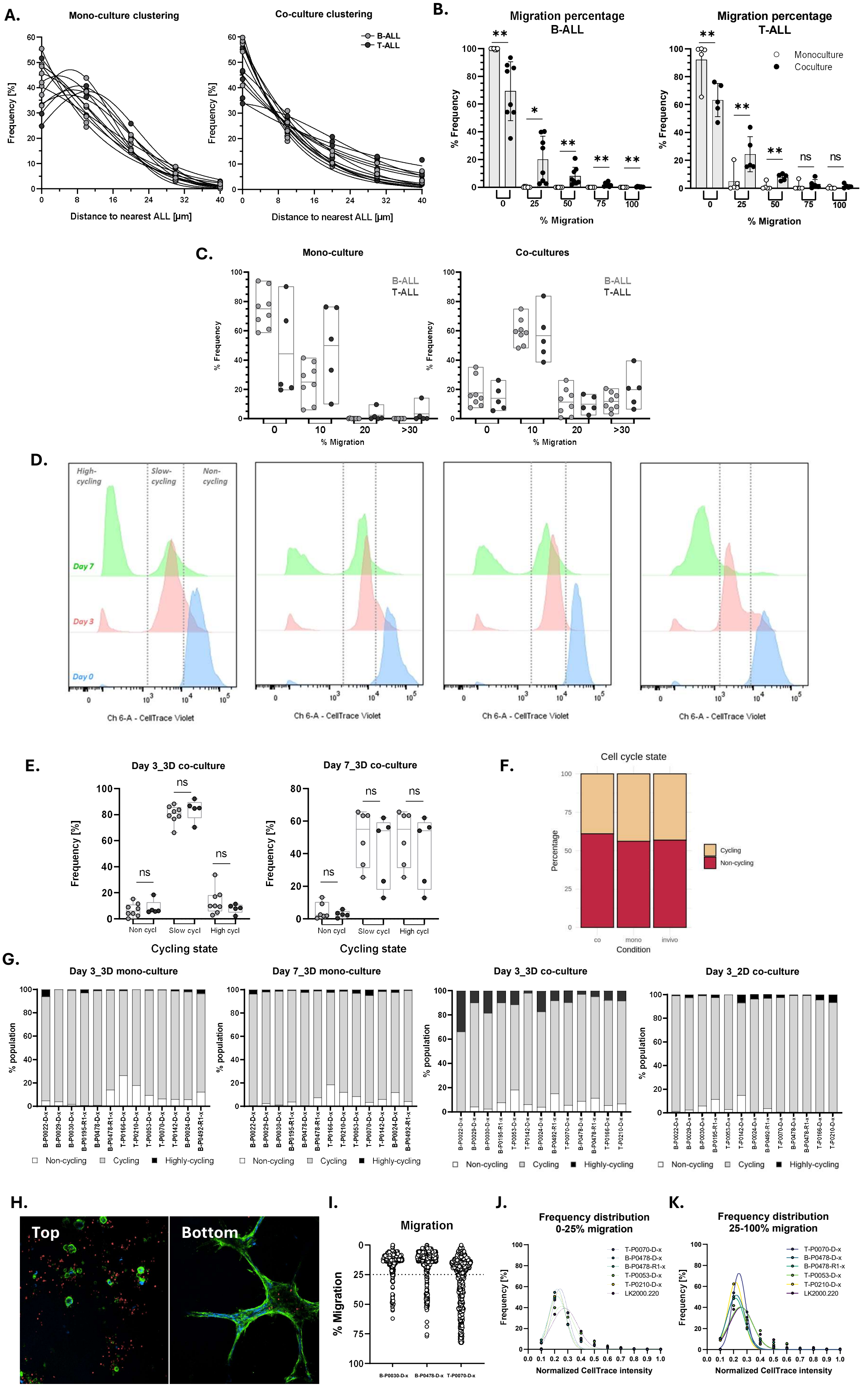
A.
Mono-culture clustering
Co-culture clustering
B-ALL
T-ALL
Frequency [%]
Distance to nearest ALL [µm]
B.
Migration percentage B-ALL
Migration percentage T-ALL
Monoculture
Coculture
% Frequency
% Migration
C.
Mono-culture
Co-cultures
B-ALL
T-ALL
% Frequency
% Migration
D.
High-cycling
Slow-cycling
Non-cycling
Day 7
Day 3
Day 0
Ch 6-A - CellTrace Violet
E.
Day 3_3D co-culture
Day 7_3D co-culture
Frequency [%]
Non cycl
Slow cycl
High cycl
Cycling state
F.
Cell cycle state
Percentage
Cycling
Non-cycling
co
mono
invivo
Condition
G.
Day 3_3D mono-culture
Day 7_3D mono-culture
Day 3_3D co-culture
Day 3_2D co-culture
% population
Non-cycling
Cycling
Highly-cycling
H.
Top
Bottom
I.
Migration
% Migration
B-P0030-D-x
B-P0478-D-x
T-P0070-D-x
J.
Frequency distribution 0-25% migration
K.
Frequency distribution 25-100% migration
T-P0070-D-x
B-P0478-D-x
B-P0478-R1-x
T-P0053-D-x
T-P0210-D-x
LK2000.220
Frequency [%]
Normalized CellTrace intensity

Suppl. Fig.4: ALL proliferation patterns and position in the hydrogel. A. Histograms of ALL aggregation measured by single-cell analysis in mono- and co-culture, with distance to nearest ALL corresponding to the minimum distances between two leukemic cells. Data are represented by triplicates for each PDX with bin center = 4. B. Subtype-based migration percentages. Left: B-ALL PDX migration, right: T-ALL PDX migration. C. Subtype based migration percentages (bin center =10). Left: Mono-culture migration, right: Co-culture migration. D. Examples of CellTrace histograms for 4 leukemic PDXs. All of the PDXs exhibit increased proliferation capability on day 7; however, their kinetics differ based on the patient. E. Subtype proliferation comparison did not reveal significant differences across the two subtypes. Cells are classified as cycling (G2M/S phase) and non-cycling (G1) phase and the corresponding percentages are plotted as a stacked bar plot across the different conditions. No significant differences can be detected, showing that the cells proliferate in a similar manner ex vivo and in vivo. G. Stacked bar plots showing the proliferation percentage for (left to right): 3D mono-culture, day 3 and day 7, 3D co-culture day 3 and 2D co-culture day 3. H. Immunofluorescence images showing the top (left) and the bottom (right) of the hydrogel (blue MSCs, green HUVECs, red ALL). I. The migration percentage of each leukemic cell was calculated based on the highest ALL position. Cells that have migration capability higher than 25% are considered cells at the bottom. J,K: Histograms showing the distribution of CellTrace intensity on the top vs bottom cell (bin center = 0.1). No significant differences are observed, indicating that there is no preferential localization based on the cycling/non-cycling state.

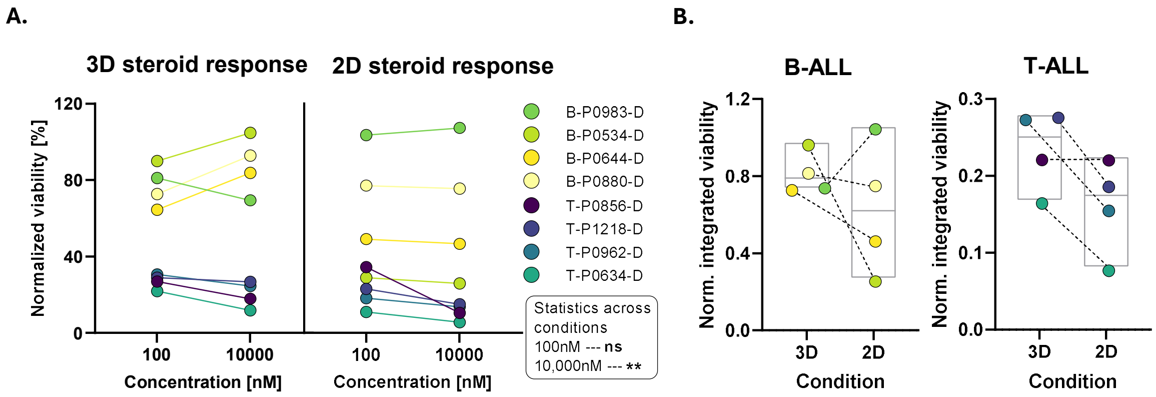

Suppl.Fig.5: Comparison of 2D and 3D response to steroid treatment, in relation to clinical outcome. A. Dot plots depicting the normalized viability upon steroid treatment in the 2D and 3D co-culture respectively. Ratio paired t test was used to evaluate statistically significant differences across the two conditions B. Subtype-based box plots indicating the patient drug response distribution in the two conditions tested, revealing increased normalized integrated viability in 3D.

Supplementary table 1: Patient-derived xenografts information.

PID	diagnosis	Disease stage	EGIL	subtype	genetics
B-P0022D-x	BCP-ALL	Diagnosis	Not available	Other	JAK2
B-P0195-R1-x	BCP-ALL	Relapse	Not available	TCF3::HLF	TCF3::HLF
B-P0029-D-x	BCP-ALL	Diagnosis	Common B	TCF3::HLF	TCF3::HLF
B-P0030-D-x	BCP-ALL	Diagnosis	Not available	TCF3::HLF	TCF3::HLF
B-P0024-D-x	BCP-ALL	Diagnosis	Not available	TCF3::HLF	TCF3::HLF
B-P0025-D-x	BCP-ALL	Diagnosis	Common B	TCF3::HLF	TCF3::HLF
B-P0485-D-x	BCP-ALL	Diagnosis	Pre-B	TCF3::PBX1	TCF3::PBX1, TP53
B-P0492-R1-x	BCP-ALL	Relapse	Pre-B	TCF3::PBX1	TCF3::PBX1
B-P0478-D-x	BCP-ALL	Diagnosis	Not available	TCF3::PBX1	TCF3::PBX1
B-P0478-R1-x	BCP-ALL	Relapse	Not available	TCF3::PBX1	TCF3::PBX1
B-P0492-R1-x	BCP-ALL	Relapse	Pre-B	TCF3::PBX1	TCF3::PBX1
B-P1050-R1-x	BCP-ALL	Relapse	Not available	BCR::ABL1	BCR::ABL1
B-P0887-R1-x	BCP-ALL	Relapse	Common B	BCR::ABL1	BCR::ABL1
B-P0887-D-x	BCP-ALL	Diagnosis	Common B	BCR::ABL1	BCR::ABL1
B-P1329-R1-x	BCP-ALL	Relapse	Common B	BCR::ABL1	BCR::ABL1, IKZF1
B-P1003D-x	BCP-ALL	Diagnosis	Pro-B	KMT2Ar	KMT2A::AFF1
B-P0974-D-x	BCP-ALL	Diagnosis	Pre-B	KMT2Ar	KMT2A::MLLT10
T-P0142D-x	T-ALL	Diagnosis	Pre- T	Other	Not available
T-P0356-R1-x	T-ALL	Relapse	Cortical-T	Other	Not available
T-P0360-R1-x	T-ALL	Relapse	Not available	Other	Not available
T-P0967-R1-x	T-ALL	Relapse	Cortical T	Other	CDKN2A, CDKN2B, NOTCH1, FBXW7
T-P0856-D-x	T-ALL	Diagnosis	Cortical T	Other	Not available
T-P1218-D-x	T-ALL	Diagnosis	Pro-T	Other	WT1, NF1, ASXL1, SETD2, BLM, EP300, AFF1, PICALM::MLLT10
T-P0053D-x	T-ALL	Diagnosis	Pre-T	Other	Not available
T-P0210-D-x	T-ALL	Diagnosis	Cortical T	Other	TLX1
T-P0166-D-x	T-ALL	Diagnosis	Cortical T	Other	Not available
T-P0070-D-x	T-ALL	Diagnosis	Cortical T	Other	Not available

Supplementary table 2: Clinical information of the patients whose PDXs were tested for dexamethasone response in the 2D and 3D platforms. From left to right: patient ID, diagnosis and subtype information, followed by the clinical outcome to the steroid prephase (d8) and response to 15 and 33 days of induction treatment (d15 and d33 respectively).

ID	***Diagnosis***	***Subtype***	***d8***	***d15***	***d33***
T-P0856-D	*T-ALL*	*Cortical*	*Poor response*	*Non-responder*	*MRD positive*
T-P1218-D	*T-ALL*	*Pro-T*	*Poor response*	*Non-responder*	*MRD positive*
T-P0962-D	*T-ALL*	*Cortical*	*Poor response*	*MRD positive*	*MRD positive*
T-P0634-D	*T-ALL*	*Mature*	*Poor response*	*Non-responder*	*MRD positive*
B-P0983-D	*BCP-ALL*	*Not available*	*Poor response*	*MRD positive*	*MRD positive*
B-P0534-D	*BCP-ALL*	*IKZF1 plus*	*Poor response*	*Non-responder*	*MRD positive*
B-P0644-D	*BCP-ALL*	*KMT2Ar*	*Poor response*	*Non-responder*	*MRD positive*
B-P0880-D	*BCP-ALL*	*Ph-like*	*Poor response*	*Non-responder*	*Non-responder*
